# First records of *Opius* and *Apodesmia* (Hymenoptera, Braconidae, Opiinae) from South Korea, with descriptions of newly-recorded species

**DOI:** 10.3897/BDJ.13.e176155

**Published:** 2025-12-26

**Authors:** So Seokho, Juhyeong Sohn, Hyojoong Kim

**Affiliations:** 1 Kunsan National University, Kunsan, Republic of Korea Kunsan National University Kunsan Republic of Korea

**Keywords:** DNA barcoding, faunistic record, natural enemy, parasitoid wasps, species distribution, systematics, taxonomy

## Abstract

**Background:**

The subfamily Opiinae comprises more than 2,000 valid species worldwide. Members of this subfamily are koinobiont endoparasitoids, with parasitism generally culminating in the eventual death of the host. Several species of Opiinae have been utilised for biological control of agricultural pests. The genus *Opius* is the largest genus within Opiinae, with more than 1,000 valid species worldwide. It is divided into several subgenera, classification of which remains under active discussion. The genus *Apodesmia* was formerly regarded as a subgenus of *Opius*, but was elevated to genus level, based on differences in the form of the occipital carina.

**New information:**

*Opius youi* Li & van Achterberg, 2013 is recorded for the first time from South Korea, representing the first record of the species outside China. *Apodesmia incisula* Fischer, 1963 is also newly recorded from South Korea, constituting the first record of the species outside Europe, where it was previously known from Germany and the Netherlands. For each species, detailed morphological descriptions are provided, accompanied by diagnostic characters illustrated with photographs of the relevant body structures. The barcode region of mitochondrial *cytochrome c oxidase I* (*COI*) was also analysed for the species.

## Introduction

The subfamily Opiinae is a relatively large taxon within the family Braconidae. Due to both its considerable size and species diversity, it has been regarded as one of the most taxonomically challenging subfamilies. More than 2,000 species of Opiinae have been recorded worldwide (Yu et al. 2016). In Korea this group was first reported by Papp (1978), with a total of 107 species currently listed in the National Species List of Korea (NIBR 2022).

The subfamily Opiinae is recognised as comprising endoparasitoids of Tephritidae and Agromyzidae (Clausen 1978, Ovruski and Schliserman 2012, Li et al. 2013). These host groups, particularly Tephritidae and Agromyzidae, are regarded as major agricultural pests. Accordingly, Opiinae species have been utilised as biological control resources against invasive fruit flies (Clausen 1978, Clarke et al. 2022). More recently, the potential of augmentative and inoculative release strategies has been evaluated (Ovruski and Schliserman 2012, Zamek et al. 2012). With the rising incidence of invasive pests facilitated by global trade, there is an increasing need to discover and document new or previously unrecorded Opiinae species that may serve as effective biological control resources.

The genus *Opius* Wesmael, 1835 represents the largest genus within Opiinae, comprising more than 1,000 described species (Fischer 1972). This genus is characterised by the following diagnostic features: the propodeum lacks a medio-longitudinal carina and is smooth or rugose; the fore wing vein m-cu is usually postfurcal and forms an angle with veins 2-CU1 and 2-M; and vein CU1b is short or absent (van Achterberg 2023).

The genus *Apodesmia* was initially classified as a subgenus of *Opius* (Fischer 1979, Tobias 1998), but including several unrelated species. Papp (2002) was the first to elevate *Apodesmia* to the genus level in the checklist, later properly based on morphological differences in the occipital carina, classification of which has since been maintained (Li et al. 2013, van Achterberg 2023). This genus is characterised by the following features: the occipital carina is curved near the mandibular base and approaches the hypostomal carina and a medio-posterior depression of the mesoscutum is present (van Achterberg 2023).

In this study, we present new morphological characters and the *COI* barcoding sequences of two newly-recorded species from South Korea. This study also provides descriptions, diagnosis and photographs of the diagnostic characters for the two species.

## Materials and methods

Materials of the Opiinae were collected using a Malaise trap. The examined specimens are deposited in Kunsan National University (KSNU). Terminology for morphological characters follows van Achterberg (1988) and van Achterberg 1993. For observation and photography, a LEICA DMC2900 digital camera mounted on LEICA M205 C microscope (Leica Geosystems AG) was used and images were stacked with Helicon software (Helicon Soft).

For DNA barcoding, genomic DNA was extracted using the LaboPass Tissue Kit (COSMOgenetech, Korea), following the manufacturer’s protocol. In order to conserve morphologically complete voucher specimens, a non-destructive extraction method (Favret 2005) in combination with the freezing method (Yaakop et al. 2009) was adapted. The modified extraction protocol was as follows: samples were incubated in 180 μl of buffer ATL and 20 μl of proteinase K without prior crushing, followed by incubation at 55°C for 10 min and subsequently stored at −22°C overnight. The extracted DNA was used to amplify the *mitochondrial cytochrome c oxidase subunit I* (*COI*) region for barcoding. Amplification was conducted with the universal invertebrate primers LCO-1490 (5′-GGTCAACAAATCATAAAGATATTGG-3′) and HCO-2198 (5′-TAAACTTCAGGGTGACCAAAAAATCA-3′) (Folmer et al. 1994). Polymerase chain reactions (PCR) were performed in 20 μl reaction volumes comprising 2 μl of DNA extract, 2 μl of primer and 16 μl of ddH₂O, using a GS1 thermo-cycler (Gene Technologies, Ltd., U.K). Cycling conditions were as follows: initial denaturation at 94°C for 1 min 30 s; 35 cycles of denaturation at 94°C for 1 min, annealing at 50°C for 1 min and extension at 72°C for 1 min; followed by a final extension at 72°C for 5 min. PCR products were visualised by electrophoresis on 1.5% agarose gels. A single band was observed and sequenced using an automated sequencer (ABI Prism 3730 XL DNA Analyzer, California, USA) at Macrogen Inc. (Seoul, South Korea). All *COI* sequences generated in this study have been deposited in GenBank. The accession numbers are as follows: PX369197 (*Opius youi*) and PX369196 (*Apodesmia incisula)*.

## Taxon treatments

### Opius youi

Li & van Achterberg, 2013

45BD84C6-4928-5CE2-837B-928B0D8CD0E1

PX369197

*Opius youi* Li & van Achterberg, 2013

#### Materials

- **Type status:** Other material. **Occurrence:** sex: female; lifeStage: adult; occurrenceID: 602C17EB-FD9D-59F0-A3B4-F71D03BB0D8D; **Taxon:** scientificName: *Opius youi* Li & van Achterberg, 2013; kingdom: Animalia; phylum: Arthropoda; class: Insecta; order: Hymenoptera; family: Braconidae; genus: Opius; specificEpithet: youi; taxonRemarks: species; **Location:** island: Jeju-do; country: South Korea; countryCode: KR; stateProvince: Jeju-si; municipality: Hangyeong-myeon; locality: Cheongsu-ri; verbatimLatitude: "33 18 13N"; verbatimLongitude: "126 14 48E"; **Event:** samplingProtocol: Malaise trap; startDayOfYear: 05 May 2020; endDayOfYear: 13 Jun 2020; year: 2020; **Record Level:** type: Dried specimen; language: English; institutionCode: NIBR, National Institute of Biological Resources

#### Description

**Figure 1.**
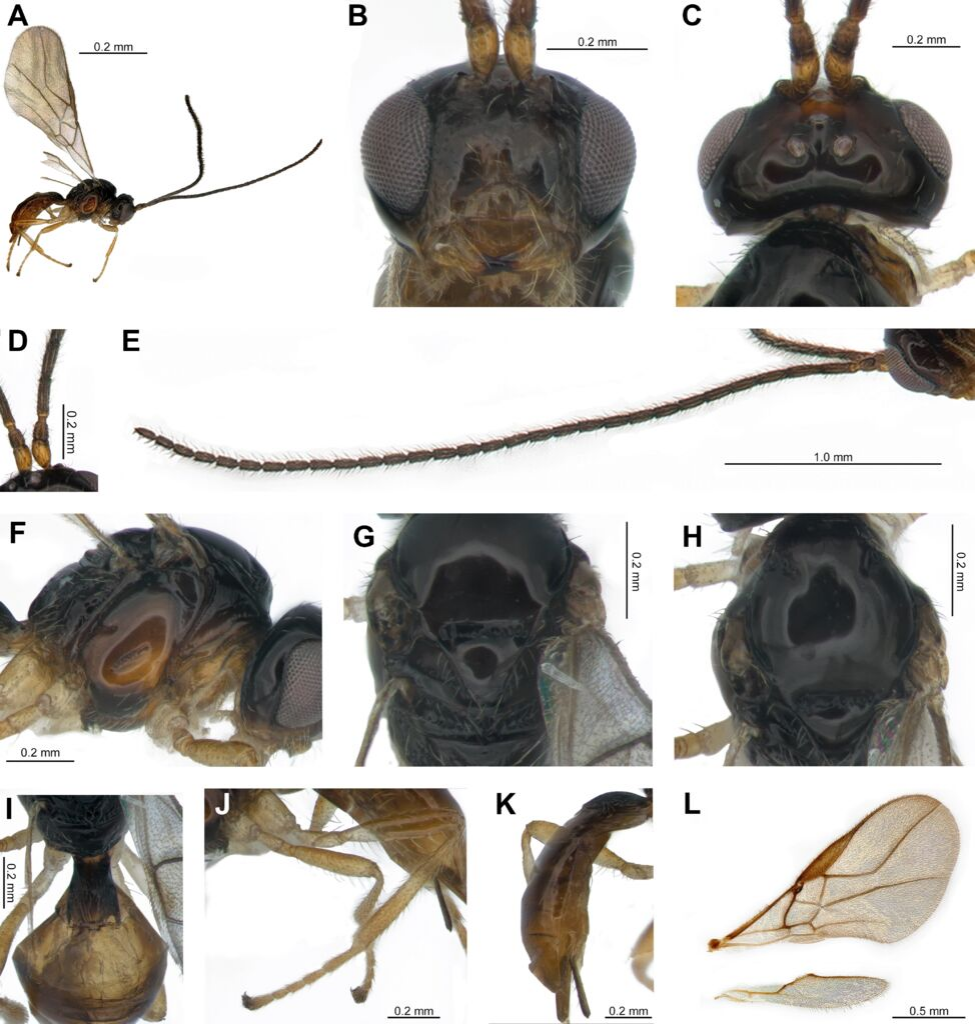
*Opius youi* Li & van Achterberg, 2013 female **A** habitus, lateral view; **B** head, front view; **C** head, dorsal view; **D** scape, pedicel, F1 and F2; **E** antennae; **F** mesosoma, lateral view; **G** mesosoma, dorsal view, scutellar sulcus; **H** mesosoma, dorsal view, mesoscutum; **I** anterior half of metasoma, dorsal view; **J** hind leg; **K** metasoma, lateral view; **L** wings.

**Female**: 1♀, Body length in lateral view 2 mm (Fig. 1A), length of antenna 3.1 mm (Fig. 1E), length of fore wing 2.5 mm (Fig. 1L).

**Colour.** Head entirely black (Fig. 1A and B). Mesosoma black, with the mesopleuron dark brown (Fig. 1F, G and H). Metasoma is black on the first tergite, becoming light brown from the second tergite onwards (Fig. 1I and K). Legs are generally yellowish, with the apices of the tarsi dark (Fig. 1J).

**Head.** Width of head 1.3 times as long as its height (Fig. 1B). Antennae consist of 31 antennomeres (Fig. 1E). Length of first flagellomere 3.1 times longer than width, length of the second 2.5 times longer than width and 0.9 times as long as the first flagellomere (Fig. 1D). First antennomere pale in comparison with third antennomere, while all flagellomeres remain dark brown (Fig. 1E). Width of the face 1.6 times as long as its height; face smooth with setose (Fig. 1B). In frontal view, gena rounded (Fig. 1B). The width of clypeus 2.2 times longer than its height (Fig. 1B). Mandible brown, becoming dark apically; widest at the base and gradually narrows towards apex (Fig. 1B). In dorsal view, length of eye 2.1 times longer than temple (Fig. 1C). Ocello-ocular line (OOL) 1.3 times as long as ocellar diameter (OD). The vertex smooth, shiny and sparsely setose (Fig. 1C).

**Mesosoma.** In lateral view, length of mesosoma 1.2 times as long as its height (Fig. 1F). The mesoscutum smooth and shiny, with the notauli distinctly impressed basally, but not extending to median area (Fig. 1H). Scutellar sulcus impressed, but lacks carinae. Scutellum smooth and shiny, bearing setae along its margins (Fig. 1G). In lateral view, mesopleuron smooth and polished. Metapleuron rugose and non-shiny, with setae present along its margins (Fig. 1F). Precoxal sulcus impressed only medially (Fig. 1F). Propodeum smooth and dull, becoming rugose near the margins; in its lower two-thirds, it curves inwards, forming a large, flattened tubercle.

**Hind leg.** Length of femur 4.4 times longer than its width (Fig. 1J). Length of tibia 8.3 times longer than width and 1.3 times as long as the femur. Length of basitarsus 3.5 times longer than wide. The coxa bears sparse setae, whereas the remaining leg segments are densely setae. Overall colouration is yellowish, with the apical parts of the tarsi darkened.

**Wing.** Length of fore wing 2.3 times longer than its width (Fig. 1L). Stigma wedge-shaped, with vein r arising from its anterior third. Proportions of vein r : 3-SR : SR1 = 1 : 15 : 31, while those of 2-SR : 3-SR : r-m = 2 : 4 : 1. Vein SR1 slightly curved dorsally at mid-length. Vein r-m is absent. Vein 1-M curved inwards at its apical three-quarters and vein 1-SR+M arises from the basal quarter of vein 1-M.

**Metasoma.** Length of first tergite 1.3 times as long as its maximum width, transversely rugose and bears a weak transverse carina medially; setae are sparsely present (Fig. 1I). Two spiracles situated dorsally. Ovipositor approximately one-quarter length of the metasoma (Fig. 1K). Hypopygium equal in length to the ovipositor and bears setae dorsally (Fig. 1K). Metasoma comprises about one-half of the total body length (Li et al. 2013).

#### Diagnosis

Notauli present only anteriorly and not reaching the median depression; the median depression is absent (Fig. 1H). In frontal view, the gena is rounded. The clypeus is flattened and comparatively large, with the median portion slightly convex dorsally (Fig. 1B). The pronotum is short. The hind tarsus is yellowish-brown, except for the telotarsus (Fig. 1J).

#### Distribution

China (Li et al. 2013), South Korea (in this study).

#### Notes

*COI* data of the original description from China and data from the present specimens (South Korea) exhibit a high degree of similarity, 99.85% of base pairs. Morphological comparison with the type material revealed differences in the head region. The most conspicuous differences occur in the relative proportions of the third and fourth antennal segments, the length of the temple and the proportions of the clypeus. Whereas the original description was based on a male specimen, the present study is based on a female, whose variation may account for the observed differences. More rigorous assessment requires re-examination of the type material and evaluation of intraspecific variation, based on a larger number of specimens. A description of the type specimen can be found in Li et al. (2013).

### Apodesmia incisula

Fischer, 1963

859BE5C1-4B14-5EAB-8942-A6DC694E236F

PX369196

*Apodesmia incisula* Fischer, 1963

#### Materials

- **Type status:** Other material. **Occurrence:** occurrenceID: 6EDB503B-5DFE-52B0-BB57-8648EDD65BA7; **Taxon:** scientificName: *Apodesmia incisula* Fischer, 1963; kingdom: Animalia; phylum: Arthropoda; class: Insecta; order: Hymenoptera; family: Braconidae; genus: Apodesmia; specificEpithet: *incisula*; scientificNameAuthorship: Fischer, 1963; **Location:** higherGeography: East Asia; island: Jeju island; country: South Korea; countryCode: KR; municipality: Jeju-si; locality: Cheongsu-ri, Hangyeong-myeon; verbatimLatitude: "33 18 13N"; verbatimLongitude: "126d 14' 48"E"; **Identification:** identifiedBy: Ju-Hyeiong Sohn; **Event:** startDayOfYear: 05 May 2020; endDayOfYear: 13 Jun 2020; year: 2020; **Record Level:** type: Dried specimen; language: English; institutionCode: NIBR, National Institute of Biological Resources

#### Description

**Figure 2.**
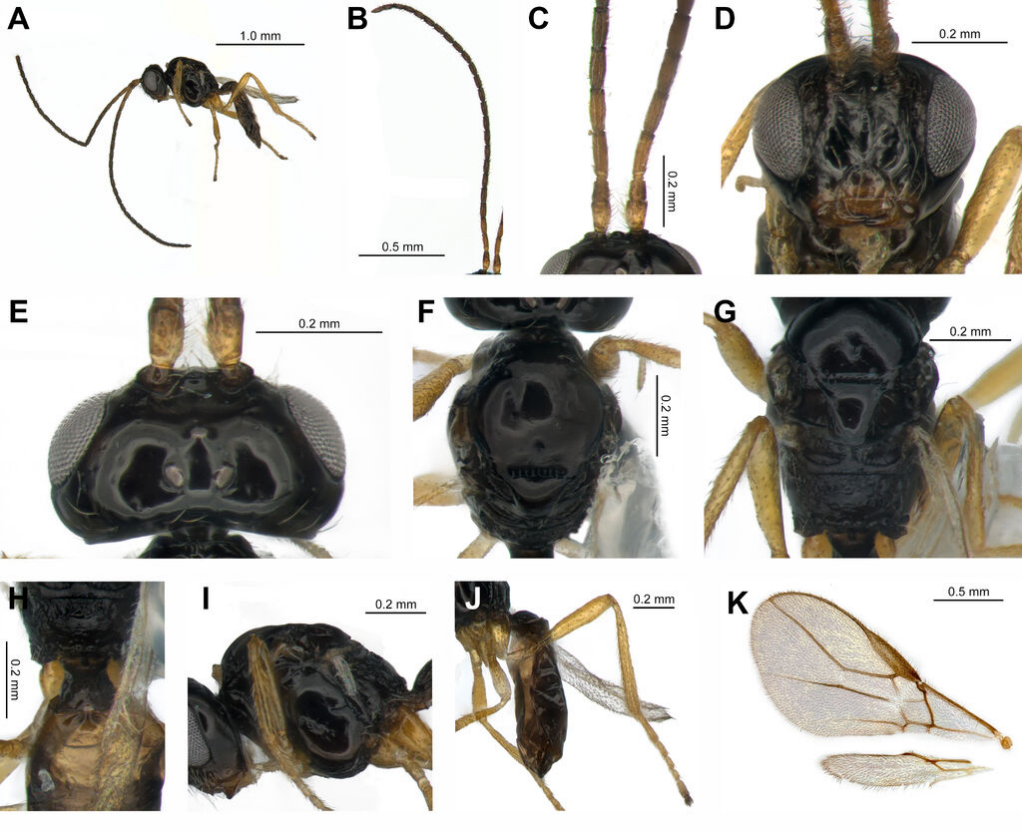
*Apodesmia incisula* Fischer, 1963 **A** habitus, lateral view; **B** antennae; **C** scape, pedicel, F1 and F2; **D** head, front view; **E** head, dorsal view; **F** mesosoma, dorsal view, mesoscutum; **G** mesosoma, dorsal view, scutellar sulcus and propodeum; **H** anterior half of metasoma, dorsal view; **I** mesosoma, lateral view; **J** metasoma and hind leg, lateral view; **K** wings.

**Male**: 1♂, Body length in lateral view 2 mm (Fig. 2A). Length of antenna 2.4 mm (Fig. 2B). Length of fore wing 2.1 mm (Fig. 2K).

**Colour**: Body and mesosoma entirely black (Fig. 2A, F, G and I). Antenna yellowish-brown up to the second flagellomere, remaining to dark brown (Fig. 2C). Legs overall yellowish-brown; apical part of tarsi, including arolium and tarsal claws, dark brown (Fig. 2J).

**Head.** Width 1.3 times as long as height in frontal view (Fig. 2D). Antenna with 25 antennomeres (Fig. 2B); length of first flagellomere 3.0 times longer than wide, its length nearly equal to that of the second flagellomere (Fig. 2C). Third flagellomere darker in colour than preceding segments, with this darker shade extending to the apical antennomeres (Fig. 2B). Width of face 1.5 times as long as its height, smooth and shiny, sparsely punctate with setae; distinct median longitudinal carina (Fig. 2D). Gena straight in frontal view. Width of clypeus 1.8 times as long as its height, semicircular in shape. Mandible yellowish-brown basally, apically darkened; basally broadened in a distinct step-like manner (Fig. 2D). In dorsal view, length of eye 1.5 times as long as temple (Fig. 2E). OOL 3.4 times longer than OD (Fig. 2E). Vertex smooth and polished.

**Mesosoma.** In dorsal view, length of mesosoma 1.6 times as long as its width (Fig. 2F); in lateral view, length of mesosoma 1.2 times as long as its height (Fig. 2I). Mesoscutum smooth and polished. Notauli present, distinctly impressed basally, thereafter faintly traceable and ending as a small pit posteriorly (Fig. 2F). Scutellar sulcus with eight carinae (Fig. 2F). In lateral view, mesopleuron smooth and polished, with few setae along the margins. Metapleuron rugose, smoother medially compared to mesopleuron (Fig. 2I). Precoxal sulcus distinct, but not extending from the anterior margin (Fig. 2I). Propodeum rugose, not dorsally curved in lateral view, 2.1 times longer than wide at maximum length; ventral margin with a carina, curved inwards at one-third of its length (Fig. 2G and H).

**Hind leg.** Length of femur 4.6 times longer than its width; length of tibia 8.8 times longer than its width; length of basitarsus 3.4 times longer than its width (Fig. 2J). Hind coxa and femur with few or no setae; hind tibia and tarsus densely setose. Hind leg overall yellowish-brown, apical part of tarsus dark brown (Fig. 2J).

**Wing.** Fore wing. Length of fore wing 2.3 times longer than its maximum width (Fig. 2K). Stigma wedge-shaped. Vein r arising from anterior third of stigma; ratio of r : 3-SR : SR1 = 1 : 7 : 15; ratio of 2-SR : 3-SR : r-m = 3 : 6 : 2. Vein SR1 shallowly curved dorsally at mid-length. Vein r-m represented only by a trace. Vein 1-SR+M arising from basal quarter of vein 1-M. Vein m-cu postfurcal.

**Metasoma.** Length of first tergite 1.3 times as long as its maximum width, medially convex and finely rugulose; dorsal carinae arising from anterior quarter of tergite and curved outwards (Fig. 2H). Second tergite anteriorly rugose towards the mid-line, otherwise smooth (Fig. 2H). Length of metasoma 0.5 times as long as body length (Fig. 2A).

#### Diagnosis

The temple is approximately two-thirds the length of the eye (Fig. 2E); the face bears a distinct median carina (Fig. 2D). The mandible is broadest at its base, broadened in a step-like manner; the gena is straight and narrowed (Fig. 2D). The notauli are distinct anteriorly, but vanish medially (Fig. 2F). The median depression is represented as a small puncture.

#### Distribution

Germany (Fischer 1963), Netherlands (Li et al. 2013), South Korea (in this study).

## Supplementary Material

XML Treatment for Opius youi

XML Treatment for Apodesmia incisula
